# Cellular Target Engagement and Dissociation Kinetics of Class I-Selective Histone Deacetylase (HDAC) Inhibitors

**DOI:** 10.3390/ijms27073036

**Published:** 2026-03-26

**Authors:** Irina Honin, Zora Novakova, Felix Feller, Simon Schneider, Linda Schäker-Hübner, Cyril Barinka, Finn K. Hansen

**Affiliations:** 1 Department of Pharmaceutical and Cell Biological Chemistry, Pharmaceutical Institute, University of Bonn, An der Immenburg 4, 53121 Bonn, Germany; 2 Institute of Biotechnology of the Czech Academy of Sciences, BIOCEV, Prumyslova 595, 252 50 Vestec, Czech Republic; zora.novakova@ibt.cas.cz (Z.N.); 3 Bonn Technology Campus, Core Facility ‘Gene-Editing’, Medical Faculty, University of Bonn, 53127 Bonn, Germany

**Keywords:** histone deacetylase (HDAC), HDAC inhibitor (HDACi), epigenetics, dissociation behavior, residence time, NanoBRET, NanoBiT

## Abstract

Histone deacetylases (HDACs) 1–3 are key regulators of gene expression and represent important therapeutic targets in cancer, neurodegenerative, and immune disorders. Many potent class I HDAC inhibitors display slow- and tight-binding kinetics, which profoundly influence their efficacy and pharmacodynamics. In particular, their dissociation rate (*off*-kinetic) is critical, since prolonged target engagement greatly influences drug efficacy in vivo. However, the *off*-kinetics of HDAC inhibitors are often overlooked in the early stages of drug development. Here, we investigated the dissociation kinetics of tucidinostat, trapoxin A, and TNG260 in comparison to the pan-HDAC inhibitor vorinostat. Using biochemical 100-fold jump dilution assays, NanoBRET assays, and cellular washout experiments, we characterized the dissociation of these compounds from purified proteins and in a cellular context. Tucidinostat showed moderately slow *off*-kinetics, while the clinical candidate TNG260 demonstrated pronounced tight-binding properties. Trapoxin A displayed remarkable discrepancies between assays, as it showed fast dissociation kinetics in the biochemical assay, but tight-binding properties in a cellular setting. These findings not only address the previously unexplored dissociation kinetics of two clinically relevant inhibitors, but also underscore the importance of comprehensive kinetic profiling of novel HDAC inhibitors in cellular models.

## 1. Introduction

Post-translational modifications (PTMs), such as acetylation, methylation, and phosphorylation of proteins, play a crucial role in human health and disease [1,2]. Among PTMs, histone acetylation is one of the most studied modifications [3,4]. The subtle equilibrium between histone acetylation and deacetylation is maintained by two opposing enzyme families. Histone acetyltransferases (HATs) catalyze the addition of acetyl groups to the ε-amino groups of lysine residues on histones, while histone deacetylases (HDACs) cleave acetyl groups [4]. Thus, un-opposed HDAC activity can lead to histone hypoacetylation, which promotes chromatin condensation, limits DNA accessibility for transcription, and ultimately results in gene-silencing effects [5,6]. Furthermore, some HDACs also remove other short acyl groups, such as butyryl or crotonyl groups, as well as fatty acids from histones and non-histone proteins [5,7,8]. Mammals express eleven zinc-dependent HDAC isoforms, which are divided into four classes and subclasses based on their phylogenetic similarity to yeast HDACs: class I (HDAC1, HDAC2, HDAC3, and HDAC8), class IIa (HDAC4, HDAC5, HDAC7, and HDAC9), class IIb (HDAC6 and HDAC10), and class IV (HDAC11) [9,10].

Among them, class I HDACs have emerged as particularly promising therapeutic targets in various diseases, including cancer, neurological diseases, infections, and inflammatory conditions [4,8,11]. Apart from HDAC8, isoforms HDAC1-3 are closely related in terms of structural similarity [1]. HDAC1 and HDAC2 share the highest sequence homology and are often redundant in biological processes [7,12]. However, they are not always functionally interchangeable. For instance, HDAC1 and HDAC2 play different roles in embryogenesis. In certain cell types only one isoform regulates specific genes (e.g., p21^CIP/Waf1^), and HDAC1 is particularly important for proper development of heart cells and neuronal proteins [13]. Since HDAC1 and HDAC2 are exclusively localized in the cell nucleus, their main substrates are histones and other nuclear proteins involved in transcriptional regulation [4]. In contrast, HDAC3 and HDAC8 are found both in nucleus and cytoplasm [4,14]. HDAC3 shares approximately 50% sequence identity with HDAC1 and HDAC2 and is involved in the circadian rhythm, energy metabolism, neuronal function, and bone remodeling [7]. Compared to HDAC1-3, HDAC8 differs considerably regarding its structure and enzymatic properties and thus only few non-histone proteins have been validated as HDAC8 substrates [7,15,16,17]. Furthermore, HDAC8 shows considerably less deacetylase activity compared to HDAC1-3 but seems to be more proficient in cleaving long-chain acyl groups [7].

Approximately 50% of the cellular HDAC1-3 enzymes are associated with multi-enzyme complexes that include other epigenetic regulators [1,4]. HDAC1 and HDAC2 are found in several repressor complexes, including repressor element-1 silencing transcription co-repressor (CoREST), nucleosome remodeling deacetylase (NuRD), SWI-independent-3A (Sin3A), and mitotic deacetylase complex (MiDAC), mesoderm induction early response (MIER), and arginine-glutamic acid dipeptide repeats (RERE) [5,18]. Although these complexes recruit the same isoforms, their biological functions are diverse [18]. In contrast, HDAC3 interacts exclusively with nuclear receptor co-repressor 1 (NCoR1) and silencing mediator of retinoic acid and thyroid hormone receptor (SMRT, also known as NCoR2) [5,14]. In the case of HDAC3, recruitment in these protein complexes, which are additionally stabilized by inositol phosphates, is essential for its enzymatic activity [1,18,19,20]. HDAC8 is the only class I isoform that functions independently without multi-enzyme complexes [14].

To date, vorinostat, belinostat, and romidepsin are approved by the Food and Drug Administration (FDA) for the treatment of malignant hematological diseases such as T-cell lymphoma, whereas panobinostat is marketed in Europe for multiple myeloma [7]. Furthermore, tucidinostat (chidamide) has been approved by the Chinese National Medical Products Administration (NMPA) for the treatment of peripheral T-cell lymphoma and advanced hormone receptor-positive breast cancer [21]. Moreover, givinostat was recently approved for *Duchenne* muscular dystrophy (DMD) [22,23]. Notably, most marketed HDAC inhibitors such as vorinostat target multiple HDAC isoforms and have displayed severe toxicities, including thrombocytopenia and fatigue, which limit their application [1,10,18]. Among the approved HDAC inhibitors, only romidepsin and tucidinostat are at least HDAC class I selective inhibitors, yet they still lack selectivity within this class and can also cause severe, dose-limiting side effects [5,16]. As a result, considerable efforts have been directed toward the development of isoform-selective HDAC inhibitors [14].

Although all Zn^2+^-dependent HDACs share a highly conserved catalytic deacetylase domain, subtle structural differences and specific substrate recognition characteristics enable the rational design of class or isoform selective inhibitors [24]. HDAC1-3 possess a characteristic “foot pocket”, which is a lipophilic internal cavity adjacent to the catalytic center [10,25]. In contrast, class IIa isoforms harbor a distinct lower pocket that can be engaged by appropriately tailored inhibitors for selective inhibition [26]. HDAC6 features a comparatively wider channel rim, which facilitates selective engagement of inhibitors with bulkier cap groups [27]. HDAC10 contains a gatekeeper residue and a sterically constricted active site that promote the binding of polyamine substrates [28]. Finally, HDAC11 functions preferentially as a fatty-acid deacylase with a high preference for long-chain acyl groups [29].

**Figure 1:**
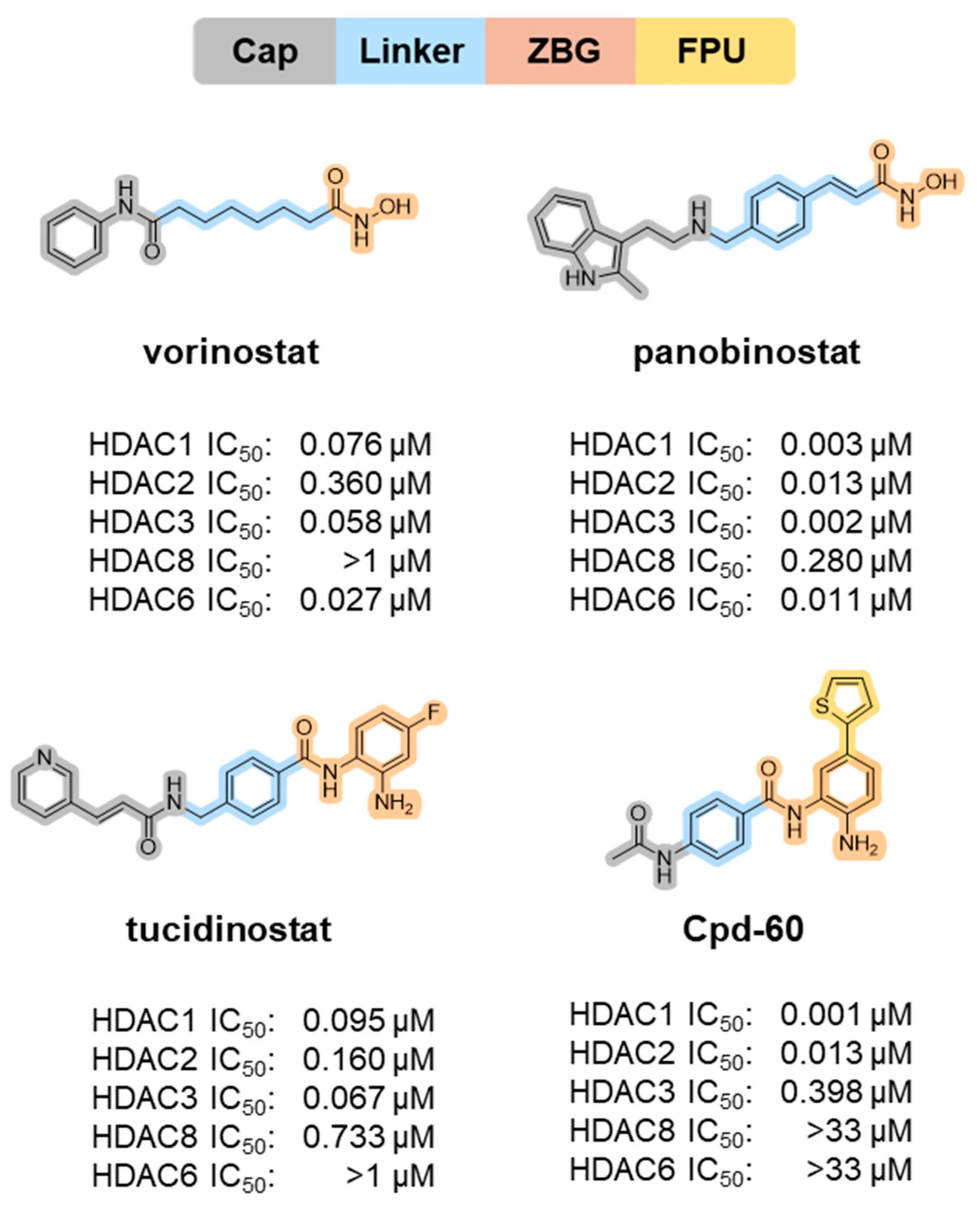
Simplified HDAC pharmacophore, the approved HDAC inhibitors vorinostat and panobinostat, and the NMPA-approved *ortho*-aminoanilide tucidinostat. FPU = foot pocket unit. Data taken from refs [5,42,43].

In general, the design of class I selective HDAC inhibitors can be easily accomplished through subtle modifications of the HDAC inhibitor pharmacophore model (see Figure 1), which is typically composed of a zinc-binding group (ZBG), which coordinates the zinc ion in the active site, a cap group, which interacts with the surface of the enzyme, and a linker. The most common ZBGs are hydroxamic acids and *ortho*-aminoanilides [30]. But HDAC inhibitors bearing a hydroxamic acid ZBG, such as vorinostat, belinostat, or panobinostat, are usually pan-HDAC inhibitors [30]. In contrast, inhibitors with an *ortho*-aminoanilide ZBG target only HDAC1-3, as exemplified by tucidinostat [5,10,18]. Other possible class I selective ZBG are alkyl-hydrazides [31], *ortho*-hydroxyanilides [32,33], thiols [5] (e.g., the active form of the prodrug romidepsin), ketones [5] (e.g., trapoxin A/B), or hydroxy-groups [5] (e.g., apicidin D2). Another approach to enhance selectivity and potency is to exploit the foot pocket [10,25]. However, similar additional cavities adjacent to the zinc ion were also reported for HDAC8 and HDAC11 [34,35,36]. In regard to HDAC1–3, the structural differences between the foot pockets of HDAC1/HDAC2 and HDAC3 offer an opportunity to achieve selectivity for HDAC1/HDAC2 over HDAC3 [1,10]. Aromatic moieties such as phenyl, thienyl, or pyridinyl are typically employed to engage the foot pocket of HDAC1/HDAC2 [4,37]. However, designing selective HDAC3 inhibitors is still challenging [30]. Thus, more recent research has focused on the selective targeting of HDAC1-3 within their respective multi-enzyme complexes [18,38,39].

Despite the progress in the development of selective class I HDAC inhibitors, most studies have primarily focused on determining the inhibitory potency between the inhibitor and the isoform to assess an inhibitor’s efficacy. Typically, parameters such as IC_50_ and *K*_i_ are used to describe how potently an inhibitor binds under equilibrium conditions [40]. Although this provides valuable initial insights, these measurements mainly capture a fixed moment of the inhibitor–target interaction in a controlled, closed system, where the concentration of the inhibitor and the target remain constant throughout the experiment [40]. However, the inhibitory potency is equally influenced by the inhibitor’s association (*k*_on_) and dissociation (*k*_off_) rate constants, and in vivo conditions differ considerably from these idealized settings [40]: in a living organism, the inhibitor concentration fluctuates dynamically due to absorption, distribution, metabolism, and elimination [40,41]. Under such circumstances, the association rate (*k*_on_) becomes less critical [40]. Instead, the dissociation behavior of the inhibitor gains more importance [41]. Tight-binding inhibitors that dissociate slowly remain bound to the target for extended periods even when systemic levels of the inhibitor decrease [40]. This prolonged target engagement results in durable pharmacological effects, allows for less frequent dosing, and potentially reduces *off*-target toxicity [8,40]. Therefore, the dissociation kinetics of an inhibitor may serve as an additional predictor for its in vivo efficacy [10,40]. In this context, selective class I HDAC inhibitors featuring an *ortho*-aminoanilide ZBG are particularly interesting, as they are known for their slow- and tight-binding properties [4]. Despite this, there is still limited information on the dissociation behavior of some otherwise well-investigated class I-selective HDAC inhibitors.

In this work, we present a comprehensive characterization of the dissociation behavior of the class I-selective HDAC inhibitors tucidinostat, TNG260, and trapoxin A compared to vorinostat. To this end, we performed different biochemical and cellular assays, including 100-fold jump dilution experiments on isolated HDAC isoforms as well as split nanoluciferase (NLuc)-based bioluminescence resonance energy transfer (NanoBRET) assays in living cells.

## 2. Results and Discussion

**Figure 2:**
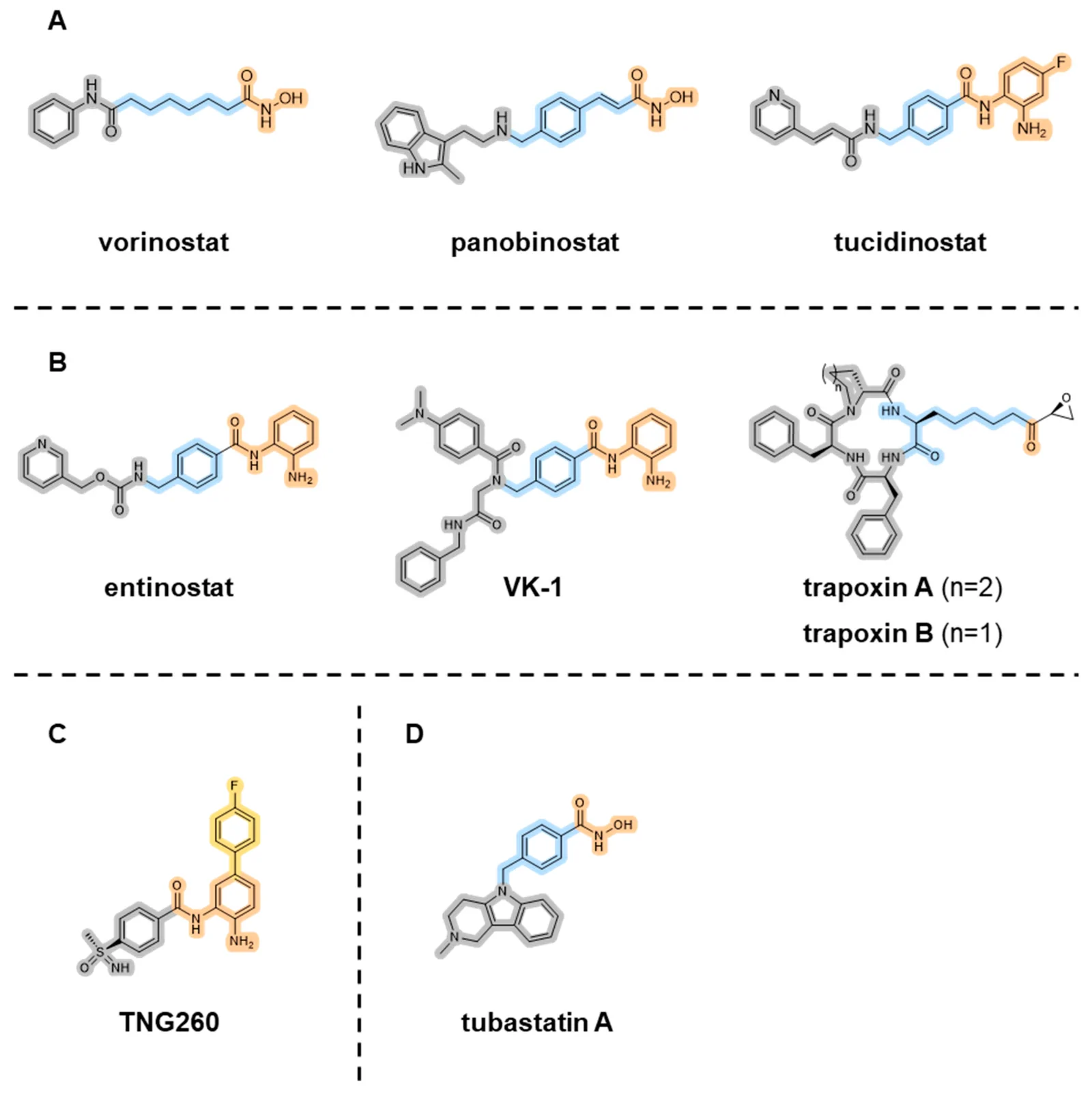
Structures of tested HDAC inhibitors. (**A**) Approved HDAC inhibitors vorinostat (pan-HDAC inhibition), panobinostat (pan-HDAC inhibition), and tucidinostat (HDAC1-3 selective). (**B**) Class I-selective HDACi entinostat, VK-1, and trapoxin A. (**C**) CoREST-selective HDAC inhibitor TNG260. (**D**) Tubastatin A is an HDAC6/10-preferential inhibitor and serves as a control.

We initially selected eight HDAC inhibitors for our investigation (see Figure 2): the approved HDAC inhibitors vorinostat, panobinostat, and tucidinostat, as well as three class I-selective HDAC inhibitors (entinostat, VK-1, and trapoxin A), the CoREST-selective inhibitor TNG260, and tubastatin A as a negative control. Vorinostat is already well characterized as a non-selective HDAC inhibitor with fast-*on*/fast-*off* properties at several isoforms [37,44]. Panobinostat is also a non-selective HDAC inhibitor. However, panobinostat is considerably more potent than vorinostat and shows isoform-dependent differences in its binding behavior [5,44]. Further, the *ortho*-aminoanilides tucidinostat, entinostat, and VK-1 are selective for HDAC1-3 but less potent than vorinostat [37]. The natural product trapoxin A possesses an epoxy ketone as ZBG and is highly potent towards class I HDACs. Additionally, we included the CoREST-selective inhibitor TNG260, which is currently being tested in a phase I clinical trial for lung cancer [45]. Interestingly, although TNG260 is under clinical investigation, there is only limited information available concerning its binding kinetics.

### 2.1. In Vitro HDAC Inhibition

First, the inhibitory activity of the selected HDAC inhibitors and controls was evaluated in a fluorescence-based biochemical HDAC inhibition assay against recombinant HDAC1, HDAC2, and HDAC3/NCoR. These measurements served as a reference for comparison with published data. As shown in Table 1, the pan-HDAC inhibitor vorinostat showed strong inhibition in a low submicromolar range at HDAC1 (IC_50_ = 0.11 µM), HDAC2 (IC_50_ = 0.21 µM), and HDAC3 (IC_50_ = 0.10 µM). Panobinostat, another pan-HDAC inhibitor, achieved single-digit nanomolar IC_50_ values at all three isoforms (HDAC1: IC_50_ = 0.0031 µM; HDAC2: IC_50_ = 0.0038 µM; and HDAC3: IC_50_ = 0.0011 µM). As expected, the HDAC6/10-preferential inhibitor tubastatin A showed markedly reduced activity against HDAC13 (IC_50_ > 5 µM), while tucidinostat showed comparable inhibitory activity to vorinostat against HDAC1-3 (HDAC1: IC_50_ = 0.24 µM; HDAC2: IC_50_ = 0.24 µM; HDAC3: IC_50_ = 0.10 µM). Additionally, the inhibitory activity of entinostat and VK-1 is listed in Table 1 as recently reported by us: compared to vorinostat, entinostat was slightly less potent at HDAC1 (IC_50_ = 0.43 µM); HDAC2 (IC_50_ = 0.35 µM); and HDAC3 (IC_50_ = 0.31 µM) [37]. In contrast, VK-1 was more preferential for HDAC1 (IC_50_ = 0.069 µM) and HDAC2 (IC_50_ = 0.13 µM) than vorinostat but showed less activity at HDAC3 (IC_50_ = 0.31 µM) [37]. In our investigation, TNG260 emerged as a potent inhibitor of HDAC1 (IC_50_ = 0.033 µM) and HDAC2 (IC_50_ = 0.086 µM) and showed particular selectivity over HDAC3 (IC_50_ = 4.5 µM). Notably, the natural product trapoxin A exhibited the highest potency, with IC_50_ values in the picomolar range (HDAC1: IC_50_ = 0.00039 µM; HDAC2: IC_50_ = 0.00027 µM; and HDAC3: IC_50_ = 0.00051 µM). Overall, the determined IC_50_ values are in good agreement with previously published data [5,37,42].

### 2.2. Investigation of Cellular Target Engagement with NanoBRET Assays

**Figure 3:**
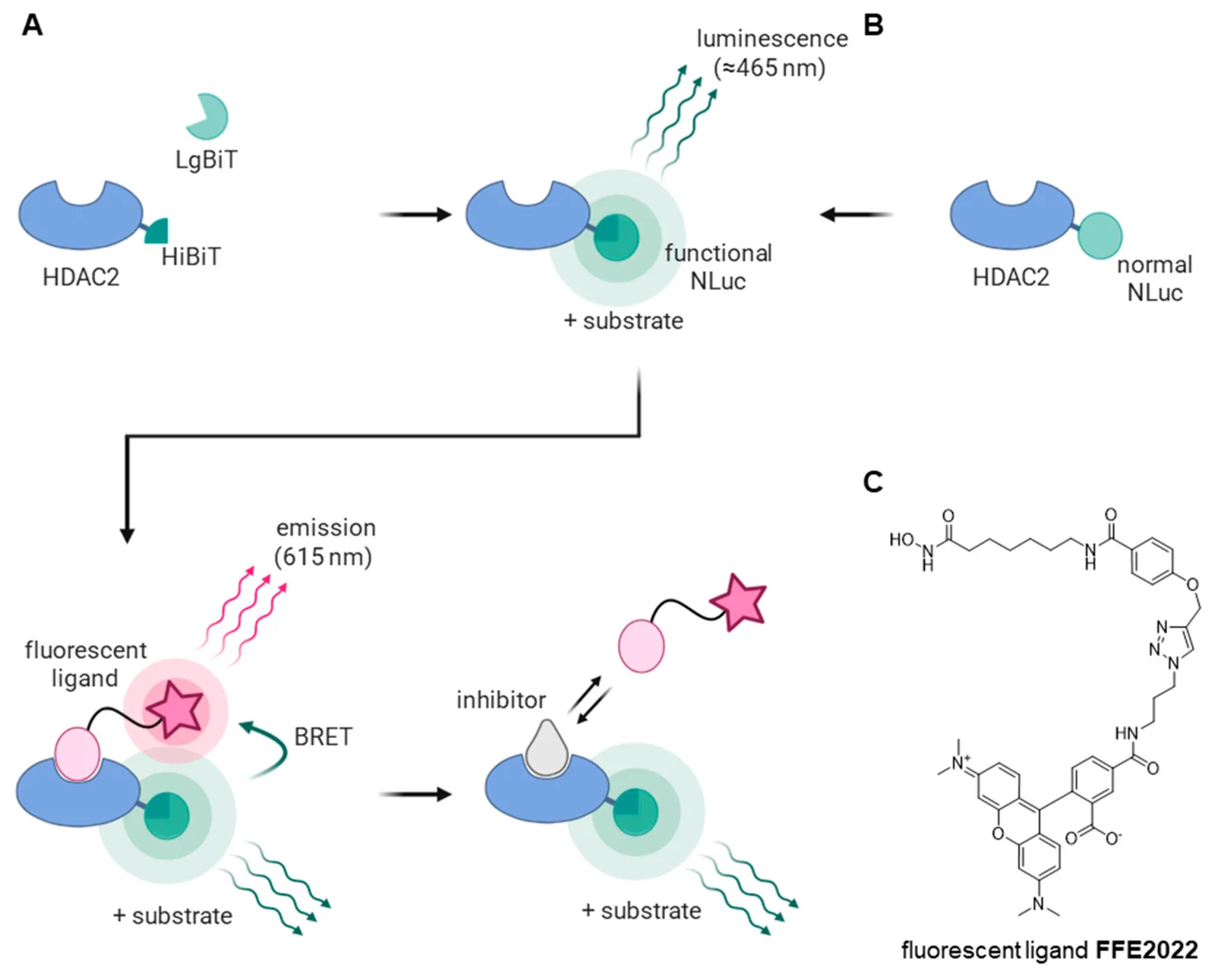
Schematic illustration of the NanoBRET assay principle using HEK293 cells with either a split NLuc (**A**) or a full-length NLuc (**B**). (**C**) Structure of the fluorescent ligand **FFE2022**.

In the next step, we performed NanoBRET assays to evaluate HDAC2 engagement in a cellular environment. NanoBRET is a proximity-based method that relies on energy transfer from a donor (NanoLuciferase-tagged target protein) to an acceptor (fluorescently labeled ligand of the target protein) through a spectral overlap [46,47]. The basic assay principle is depicted in Figure 3. For this study, we employed a split NanoLuciferase (NLuc) as the energy donor, which consists of a large binary technology subunit (LgBiT) and a small high-affinity binary technology (HiBiT) subunit [47]. When these subunits bind to each other, they reconstitute a functional NLuc, which emits bioluminescent light upon the addition of a substrate (see Figure 3A) [47]. The complementation occurs with a high binding affinity in the picomolar range, ensuring a stable and sensitive signal [47]. For this experiment, we acquired HEK293 cells, which stably express the LgBiT subunit of NLuc. Furthermore, we fused the HiBiT subunit to the C-terminus of HDAC2 via CRISPR-Cas9 genome editing. This approach offers two key advantages: the small HiBiT-Tag only minimally modifies HDAC2, and the level of HDAC2 in the cells remains largely unchanged [48,49]. In theory, the HiBiT–LgBiT technology should preserve the ability of HDAC2 to function as the catalytic subunit of multiprotein complexes, allowing inhibitors to be tested under near-native cellular conditions [49,50]. This is especially important since the selectivity of HDAC inhibitors within class I seems to be highly dependent on the multiprotein complex, such as CoREST, NuRD, Sin3A, MIER, NCoR, etc., to which the respective HDAC enzyme is bound [1,39,51,52].

We recently reported a similar NanoBRET assay setup to study HDAC6 target engagement in HeLa cells [49]. As in our previous work, we used the fluorescent ligand **FFE2022**, which is a TAMRA-labeled vorinostat derivative with a short propyl linker (Figure 3C). In LgBiT-expressing HEK293^HDAC2-HiBiT^ cells, the fluorescent ligand **FFE2022** displayed a *K*_D_ value of 0.15 µM (Figure S1, Supporting Information), which was comparable to the previously reported *K*_D_ value for HDAC6 in LgBiT-expressing HeLa^HDAC6-HiBiT^ cells [49]. To assess the cellular permeability of the fluorescent ligand in HEK293 cells, cells were treated with the detergent digitonin (50 ng/µL), which selectively disrupts the plasma membrane, while leaving nuclear and mitochondrial membranes intact [53]. The *K*_D_ value in permeabilized cells (*K*_D_ = 0.097 µM) was not substantially different from the *K*_D_ in intact cells (Figure S1, Supporting Information), which confirms that the tracer sufficiently permeates the plasma membrane in HEK293 cells.

For the displacement setup, cells were incubated with the fluorescent ligand **FFE2022** at a final concentration of 0.5 µM, yielding approximately 80% occupancy of HDAC2. Additionally, varying concentrations of different HDAC inhibitors were added. In a competitive binding setup, the displacement of the fluorescent ligand by unlabeled inhibitors led to a reduction in the BRET signal, indicating target engagement (see Figure 3, bottom). The results of the cellular target engagement studies are summarized in Table 1 and Figure S2 (Supporting Information). For vorinostat, a submicromolar EC_50_ value of 0.38 µM was determined. Particularly strong inhibition was observed for panobinostat (EC_50_ = 0.0034 µM), trapoxin A (EC_50_ = 0.00037 µM), and TNG260 (EC_50_ = 0.012 µM). Tubastatin A (EC_50_ = 1.7 µM), entinostat (EC_50_ = 1.9 µM), and tucidinostat (EC_50_ = 1.4 µM) exhibited similar EC_50_ values at HDAC2. However, while tucidinostat showed comparable inhibition of HDAC2 (IC_50_ = 0.24 µM) to vorinostat (IC_50_ = 0.21 µM) in the biochemical HDAC inhibition assay, it performed markedly worse than vorinostat in the NanoBRET assay. The reduced activity of entinostat and tucidinostat in the cellular NanoBRET assay, compared with the biochemical HDAC2 assay, may be attributable to limited cellular permeability, potential efflux mechanisms, complex selectivity profiles, or non-specific binding. Similarly, VK-1 (EC_50_ = 0.74 µM) also demonstrated lower potency compared to vorinostat in the NanoBRET assay, which might be attributed to its physicochemical properties or slow-binding characteristics. Interestingly, tubastatin A appeared to be more potent against HDAC2 in cells compared to the biochemical assay.

For further studies, we focused on inhibitors, whose *off*-kinetics have so far been scarcely studied. Previously, Moreno-Yruela et al. reported that panobinostat’s dissociation behavior properties differ depending on the isoform: it binds tightly to HDAC2 and shows fast dissociation kinetics at HDAC1 and HDAC3 [44]. Entinostat is also well-characterized: it displays moderate dissociation kinetics at HDAC1 [37,54] and binds tightly to HDAC2 [54] and HDAC3 [37]. VK-1 has similar properties to entinostat at HDAC1 and HDAC3 [37]. Trapoxin A has been previously described with exceptionally tight-binding properties for class I HDACs [44,55]. However, earlier studies assessed its dissociation behavior only indirectly through the determination of the binding mode at HDAC1 and HDAC2 [44] or without specification of the isoform [56]. To date, direct dissociation measurements have been limited to HDAC8 [55]. Notably, tight-binding properties at HDAC1 have only been demonstrated for trapoxin B, which is an analog of trapoxin A with a pyrrolidine ring instead of a piperidine ring (see Figure 2B) [57]. Interestingly, the dissociation behavior of tucidinostat and TNG260 has also not been reported yet. Thus, we focused on tucidinostat, trapoxin A, and TNG260, as well as vorinostat and tubastatin A as controls in the following experiments.

### 2.3. Comparison of Split NLuc-Based and Conventional NanoBRET Assays

However, before performing dissociation experiments, we examined whether data from a split NLuc-based NanoBRET assay are comparable to those from a conventional NanoBRET assay. In a conventional NanoBRET assay, cells are typically transfected with expression plasmids encoding the target gene fused to a full-length NLuc [46,47,50,58]. These plasmids are usually driven by strong promoters, which lead to an overexpression of the tagged target protein [48,50,59]. The imbalance of protein levels may cause disadvantages such as protein aggregation, malfunction, ectopic localization, or formation of non-functional complexes [50,59,60]. As mentioned before, in our split NLuc-based NanoBRET assay, the HDAC2 level is not substantially altered [48,49]. To generate a full-length HDAC2-NLuc HEK293T cell line, we stably transfected HEK293T cells with plasmids carrying HDAC2-NLuc fusion genes in which NLuc was fused to the C-terminus of HDAC2 (Figure S3, Supporting Information). Furthermore, we acquired data with HDAC1-NLuc cells, which were already reported in previous studies by Ptacek et al. [61] The EC_50_ values are summarized in Table 2. For HDAC1, the observed trends from the conventional NanoBRET assay were consistent with the biochemical assay in most cases. Interestingly, tucidinostat (EC_50_ = 18 µM) and tubastatin A (EC_50_ = 72 µM) stand out with comparatively high EC_50_ values at HDAC1 in the NanoBRET assay, but the overall potency trend relative to the reference compound vorinostat remains consistent. At HDAC2, a similar trend was observed. In the case of trapoxin A (HDAC2: IC_50_ = 0.00027 µM; HDAC2-HiBiT: EC_50_ = 0.00037 µM; HDAC2-NLuc: EC_50_ = 0.00032 µM) and TNG260 (HDAC2: IC_50_ = 0.086 µM; HDAC2-HiBiT: EC_50_ = 0.012 µM; HDAC2-NLuc: EC_50_ = 0.045 µM;), the results of the biochemical HDAC assay and the NanoBRET assays (split NLuc or a full-length NLuc) agree with each other very well. However, we noticed differences in the observed potency for tucidinostat and trapoxin A. Tucidinostat showed an EC_50_ of 0.69 µM at HDAC2-NLuc, comparable to vorinostat (EC_50_ = 0.98 µM). In contrast, in the split NLuc-based NanoBRET, tucidinostat was considerably less potent (EC_50_ = 1.4 µM) than vorinostat (EC_50_ = 0.38 µM). Furthermore, tubastatin A showed striking differences: Its EC_50_ value in HDAC2-NLuc cells is considerably higher than in HDAC2-HiBiT cells (EC_50_ = 21 µM vs. EC_50_ = 1.7 µM). Notably, the observed potency of tubastatin A in HDAC2-HiBiT cells aligns with the findings of other studies reporting the effects of tubastatin A on class I inhibition marker acetylated histone H3 at single-digit micromolar concentrations [62,63]. These results indicate that the split NLuc-based NanoBRET assay reflects cellular target engagement more accurately than conventional NanoBRET assays.

### 2.4. Characterization of the Dissociation Behavior

Next, we evaluated the dissociation behavior of the selected HDAC inhibitors using 100-fold jump dilution experiments, cellular NanoBRET residence time assays, as well as cellular wash-out experiments.

**Figure 4:**
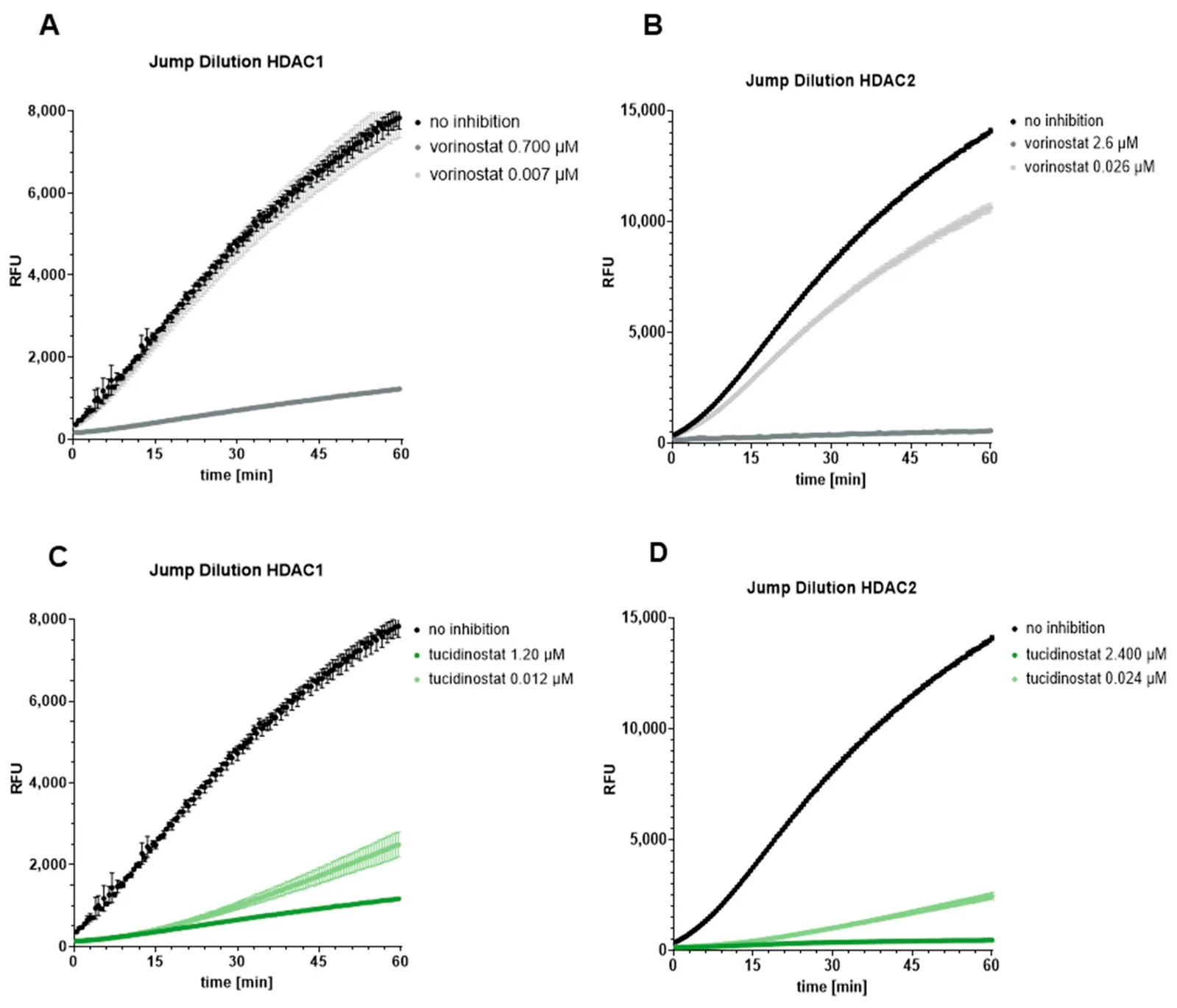

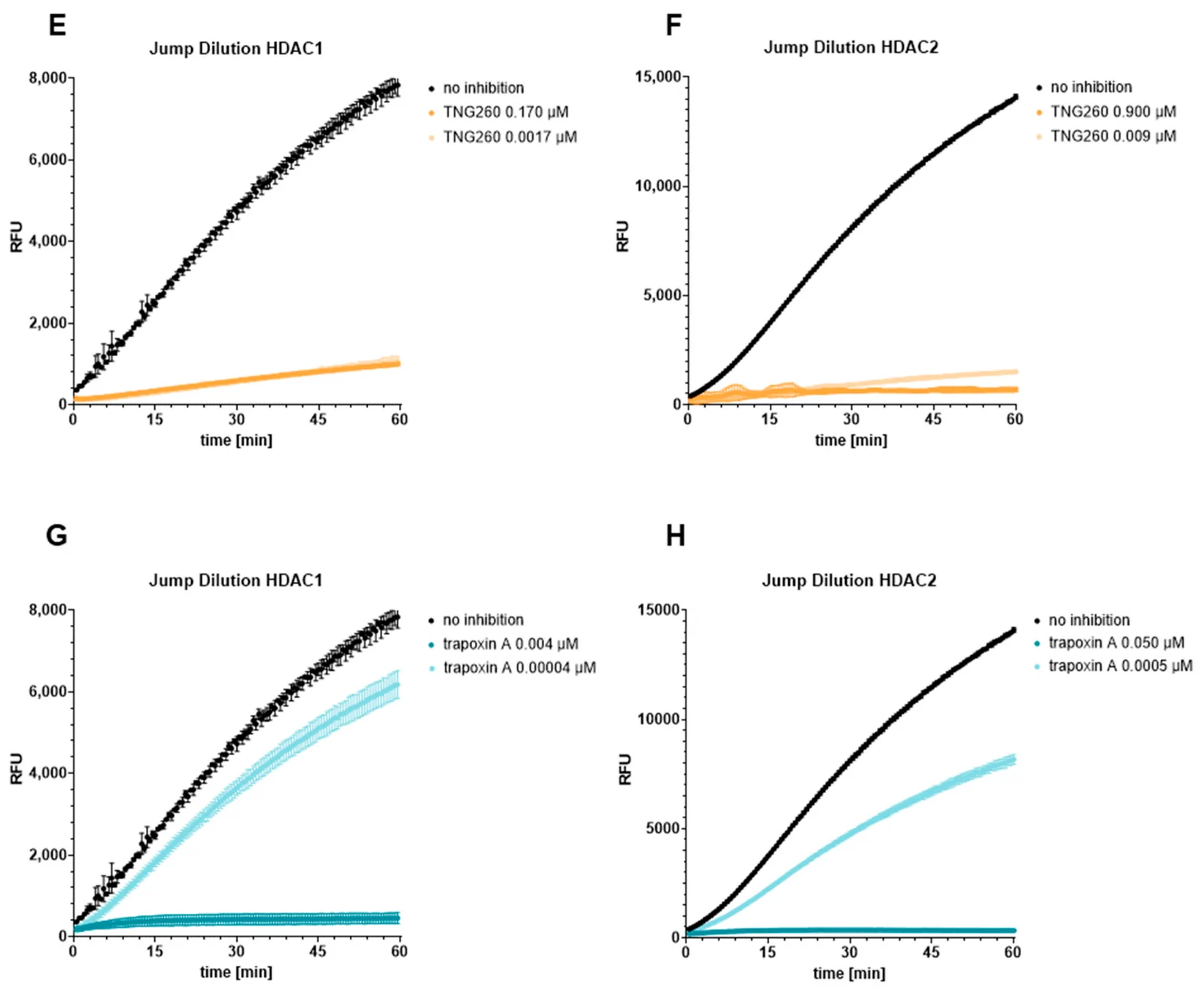
Analysis of the dissociation behavior of vorinostat (**A**,**B**), tucidinostat (**C**,**D**), TNG260 (**E**,**F**), and trapoxin A (**G**,**H**) at HDAC1 (**left**) and HDAC2 (**right**). Apart from trapoxin A, the employed inhibitor concentrations were set to 7.5-fold (HDAC1) and 10-fold (HDAC2) of their respective IC_50_ values on the corresponding enzyme and are indicated in the graph. For trapoxin A the concentrations were set to 15-fold (HDAC1) and 20-fold (HDAC2) of its IC_50_. Representative curves from at least two independent experiments are shown.

In jump dilution experiments, the respective purified HDAC isoforms were pre-incubated for 1 h with a test compound at a concentration that is a multiple of its IC_50_ value in an “incubation mix”. Then the “incubation mix” was rapidly diluted 1:100, and the dissociation of the test compounds was continuously monitored for 60 min. The results are summarized in Figure 4. We used the fast-*on*/fast-*off* HDAC inhibitor vorinostat as a control, and as expected, vorinostat rapidly dissociated from HDAC1 and HDAC2 (see Figure 4A,B) [37]. Tucidinostat dissociated from HDAC1/HDAC2 at a moderate rate (Figure 4C,D). In contrast, TNG260 maintained the inhibition of HDAC1 and HDAC2 after dilution, which indicates tight binding properties (Figure 4E,F). Surprisingly, trapoxin A showed fast-*off* kinetics at HDAC1 and HDAC2 in this assay setup, although previous reports described it as a tight-binding inhibitor for class I HDACs [44,55,56].

**Figure 5:**
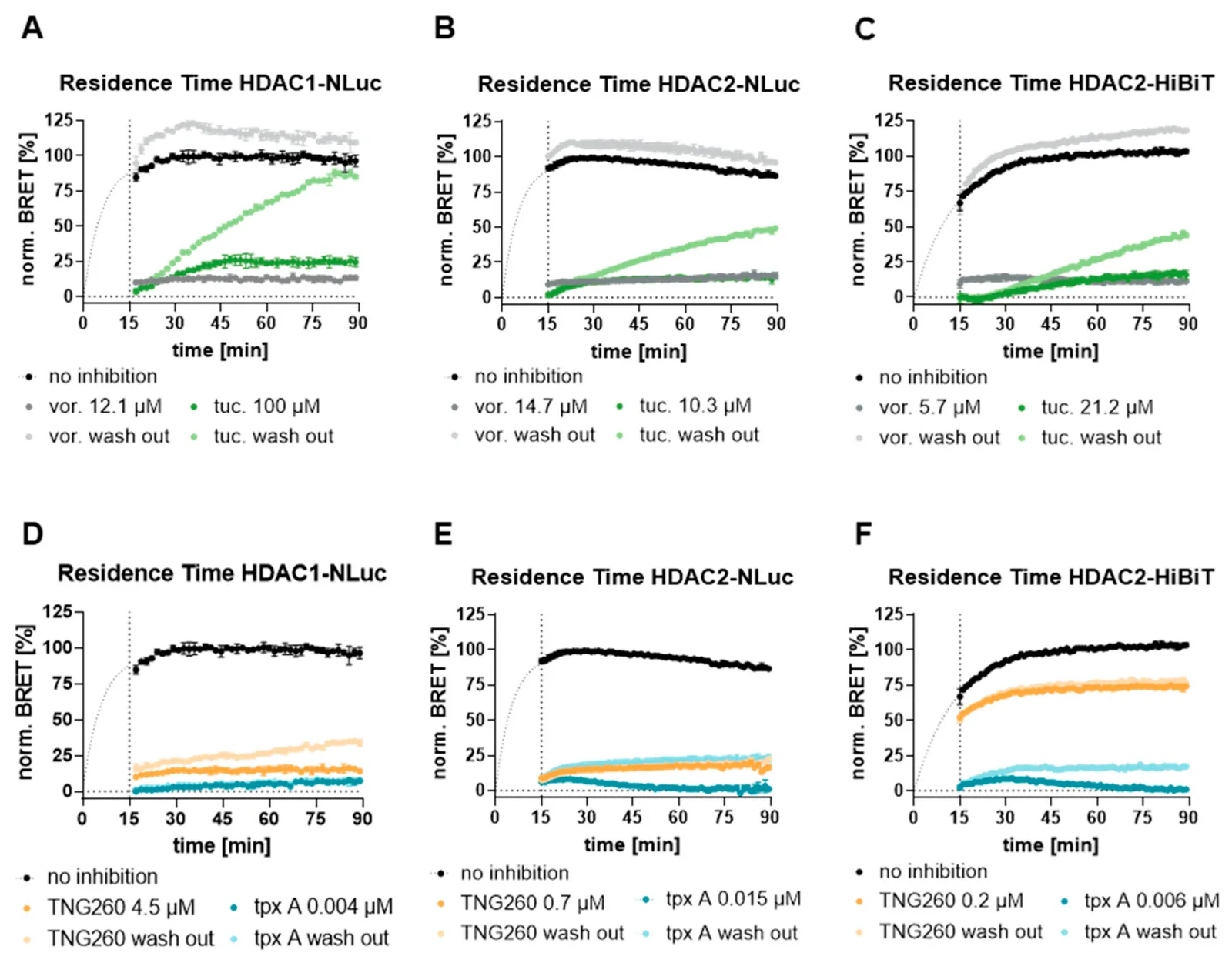
Intracellular residence time of HDAC inhibitors vorinostat (vor.; gray; (**A**–**C**)), tucidinostat (tuc; green; (**A**–**C**)), TNG260 (orange; (**D**–**F**)), and trapoxin A (tpx A; blue; (**D**–**F**)) in HDAC1-NLuc cells (**left**), HDAC2-NLuc cells (**middle**), and HDAC2-HiBiT cells (**right**). Employed inhibitor concentrations are set to a multiple of their respective EC_50_ values in the NanoBRET assay and are indicated in the graph. The maximum applied concentration was capped at 100 µM. Data are normalized to the maximum signal of DMSO-treated cells and the lowest signal (trapoxin A, no washout). The start of the fluorescence measurement (t = 15 min) after removing unbound inhibitor (t = 0 min) with assay buffer, the un-shifted abscissa (normalized BRET ratio = 0), and the presumed course of the vehicle’s BRET ratio are indicated as dashed lines. Representative curves from at least two independent experiments are shown.

Next, we performed NanoBRET residence time assays according to the protocol of Robers et al. with minor modifications [46,58]. The results are shown in Figure 5. Briefly, cells were first incubated with an inhibitor concentration set to a multiple of the respective EC_50_ value (for details, see Figure 5). After inhibitor removal and several washing steps, the fluorescent ligand **FFE2022** was added at a saturating concentration. In addition, we permeabilized the cells by treatment with digitonin (50 ng/µL) to ensure that the dissociation kinetics are not influenced by intracellular trapping of the inhibitors [46]. Changes in the BRET signal were monitored for 90 min. An increase in the BRET signal is indicative of binding of the fluorescent ligand, which can only occur when the tested inhibitor dissociates. Again, we compared results from HDAC1-NLuc cells, HDAC2-NLuc cells, and HDAC2-HiBiT cells; however, all cell lines showed the same trends. As expected, vorinostat dissociated near-instantaneously from HDAC1 and HDAC2. Tucidinostat dissociated slowly from HDAC1 and HDAC2 at a more moderate rate, with dissociation from HDAC1 occurring slightly faster. In contrast to the results of the jump dilution experiments, but in agreement with previous reports, trapoxin A bound tightly to HDAC1 and HDAC2 in a cellular environment (see Figure 5D–F). This remarkable discrepancy raises the question which of these assays, and which assay formats in general, is predictive of the binding kinetics of trapoxin A in vivo. As expected, TNG260 was also found to be a tight-binding HDAC1 and HDAC2 inhibitor. Interestingly, we observed differences for TNG260 in HDAC2-HiBiT cells and HDAC2-NLuc cells: in HiBiT cells, HDAC2 could not be fully inhibited by TNG260. However, since the curves of washed and unwashed cells are nearly congruent, we still identified TNG260 as a tight-binding inhibitor. We attribute this phenomenon to the distinct states of HDAC2, either as free enzyme or as part of multiprotein complexes in a cellular environment: Previous studies have shown that HDACs dynamically assemble and disassemble into multiprotein complexes [1] and that *ortho*-aminoanilides preferentially bind free HDACs over complex-bound HDACs [1,51,52]. In addition, it has been shown that the cellular regulator InsP6 stabilizes HDAC complexes and affects both potency and selectivity of *ortho*-aminoanilide HDAC inhibitors towards these complexes [1,51]. In particular, *ortho*-aminoanilides containing a FPU become trapped in these inactive complexes leading to long-lasting inhibition, whereas non-selective or non-FPU analogs behave differently [1]. Reportedly, TNG260 selectively targets HDAC1 and HDAC2 in CoREST multiprotein complexes with comparable potency, while exhibiting no activity up to 100 µM against NCoR, NuRD, and Sin3 complexes [39]. We therefore hypothesize that in the HDAC2-HiBiT cells, HDAC2 predominantly exists within multiprotein complexes, of which only the CoREST complex is susceptible to inhibition by TNG260. Consequently, total inhibition of HDAC2 is not achievable. In contrast, in HDAC2-NLuc cells, HDAC2 is strongly overexpressed, which likely results in a substantial pool of free enzyme that can be efficiently targeted by TNG260, resulting in full inhibition. In light of these considerations, the observed discrepancy in dissociation behavior for trapoxin A between the biochemical assay on the isolated enzyme and the cellular assays may also arise from distinct conformational or complex-associated states of class I HDACs in cells. An explanation might be that trapoxin A stabilizes a complex-bound enzyme state and remains transiently retained within the complex, despite its fast dissociation from the isolated enzyme.

**Figure 6:**
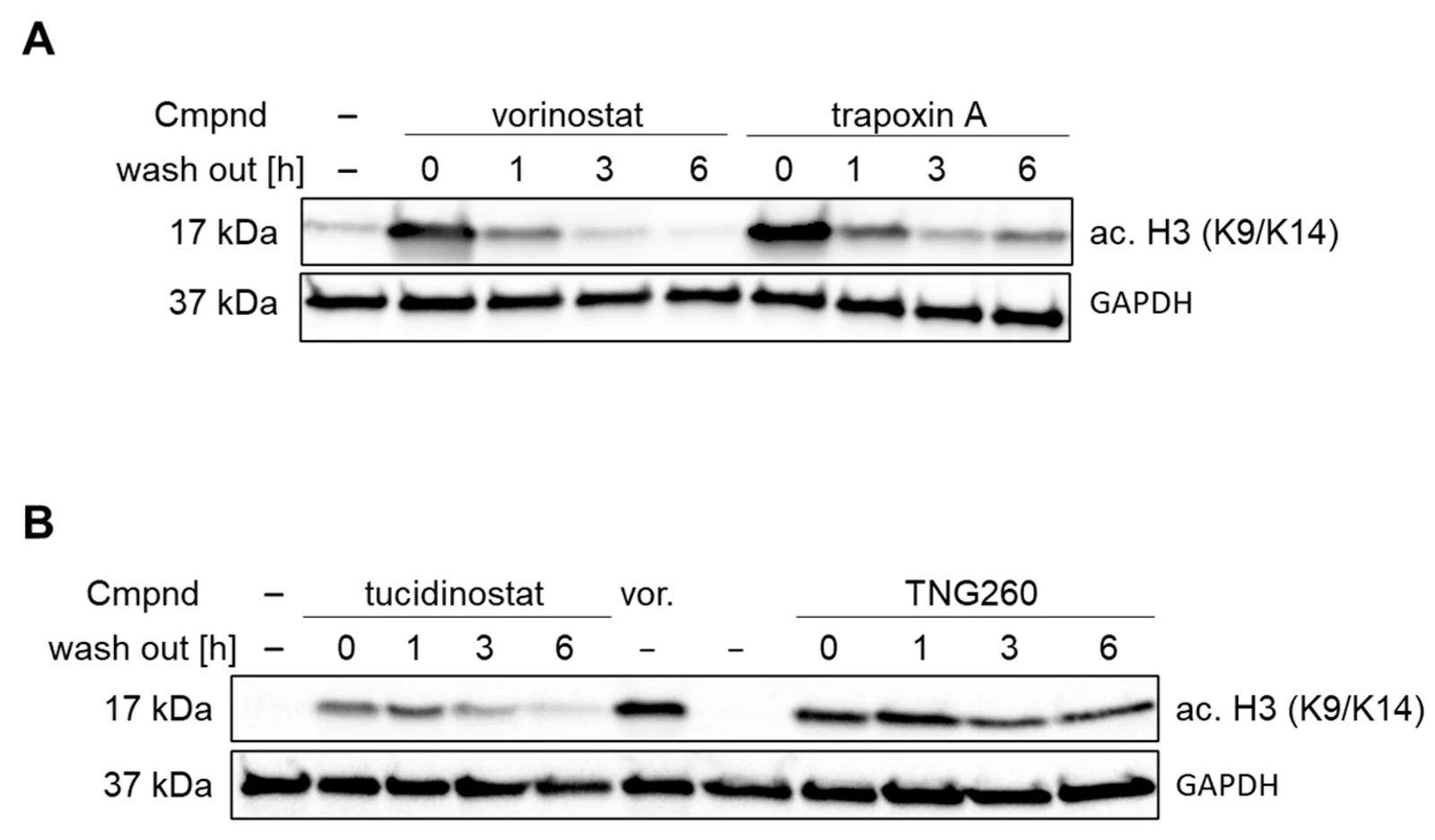
Immunoblot analysis of HEK293 cell lysates. Protein levels of acetylated histone H3 after washout experiments are shown. After incubation with vehicle (DMSO) or 10 µM of vorinostat (24 h), trapoxin A (24 h), tucidinostat (72 h), or TNG260 (72 h), cells were washed and further incubated for 1 h, 3 h, or 6 h in fresh cell culture medium without inhibitor. (**A**) Wash out experiments after treatment with vorinostat (vor.) or trapoxin A. (**B**) Wash out experiments after treatment with tucidinostat or TNG260. Representative images from at least two replicates are shown. DMSO (0.025%) was used as vehicle control and GAPDH as loading control.

To validate the results of the NanoBRET residence time assay, we performed wash out experiments using acetyl histone H3, a known substrate of class I HDACs, as a readout (see Figure 6 and Figure S4 in the Supporting Information). In short, native HEK293 cells were incubated with vorinostat, trapoxin A, tucidinostat, or TNG260. After incubation with the respective inhibitor, cells were washed to eliminate residual inhibitor, and fresh inhibitor-free medium was added. The cells were then further incubated for 1, 3, or 6 h before lysis. This approach allowed us to assess the persistence of HDAC inhibition following compound removal. In the case of vorinostat, histone H3 acetylation was barely visible after 6 h of wash out, confirming its rapid dissociation from class I HDACs, as previously observed in jump dilution experiments and the NanoBRET assays. For tucidinostat, histone H3 acetylation was still weakly detectable after 6 h, indicating that it dissociates more slowly than vorinostat. Trapoxin A had a more durable effect on histone H3 acetylation than tucidinostat. However, since histone H3 acetylation was diminished noticeably after 6 h, trapoxin A was also clearly washed out. In contrast, TNG260 maintained high histone H3 acetylation levels even at 6 h, consistent with the tight-binding behavior observed in jump dilution experiments and the NanoBRET assay. In summary, these results confirm the findings from the NanoBRET residence time assay.

## 3. Materials and Methods

### 3.1. Cell Culture

LgBiT-expressing HEK293 cells (Promega, #N267A) were cultured in DMEM medium (PAN Biotech GmbH; Aidenbach, Germany) supplemented with 10% fetal bovine serum (PAN Biotech GmbH; Aidenbach, Germany), 4 mM L-glutamine (PAN Biotech GmbH; Aidenbach, Germany), 1 mM sodium pyruvate (ThermoFisher Scientific Inc.; Waltham, MA, USA), 100 IU/mL penicillin, and 0.1 mg/mL streptomycin (PAN Biotech GmbH, Aidenbach, Germany). HEK293T cells were cultured in DMEM medium (Sigma Aldrich, St. Louis, MO, USA) supplemented with 10% fetal bovine serum (Sigma Aldrich, St. Louis, MO, USA) and 4 mM L-glutamine (PAN Biotech GmbH; Aidenbach, Germany). Both cell lines were cultivated at a humidified atmosphere at 37 °C containing 5% CO_2_. Mycoplasma contamination was routinely excluded by PCR.

### 3.2. HDAC Inhibition Assay

The HDAC inhibition assay was performed according to a previously published protocol [37]. Test compounds were serially diluted (1:3) in assay buffer (50 mM Tris-HCl, pH 8.0, 137 mM NaCl, 2.7 mM KCl, 1 mM MgCl_2_, 0.1 mg/mL BSA (all components: Sigma-Aldrich/Merck KGaA, Darmstadt, Germany)). Subsequently, 5 µL of these serial dilutions were added to 25 µL assay buffer into black 96-well microplates (OptiPlate-96 Black, Revvity, Lübeck, Germany; #6055270). Next, 10 µL of human recombinant HDAC1 (BPS Bioscience Inc., San Diego, CA, USA, #50051), HDAC2 (BPS Bioscience Inc., San Diego, CA, USA; #50052) or HDAC3/NCoR (BPS Bioscience Inc., San Diego, CA, USA; #50053) in assay buffer were added. Following an incubation period of 60 min at room temperature, 10 µL of the fluorogenic substrate ZMAL [64] (Z-Lysin(Ac)-AMC) were added and the plate was incubated for additional 90 min at 37 °C. Finally, 50 µL trypsin (Sigma-Aldrich/Merck KGaA, Darmstadt, Germany) in trypsin buffer (0.4 mg/mL in 50 mM Tris-HCl, pH 8.0, 100 mM NaCl (all components: Sigma-Aldrich/Merck KGaA, Darmstadt, Germany)) was added, followed by 30 min incubation at 37 °C. The final assay volume of 100 µL contained the following enzyme concentrations: 140 pg/µL HDAC1, 95 pg/µL HDAC2, and 100 pg/µL HDAC3/NCoR. Fluorescence (excitation: 355 nm, emission: 460 nm) was measured using a FLUOstar OPTIMA microplate reader (BMG LABTECH GmbH, Ortenberg, Germany). IC_50_ values were determined from normalized dose–response curves fitted with a three-parameter logistic model (GraphPad Prism 8.0, San Diego, CA, USA). All compounds were tested in duplicates and IC_50_ values represent means from at least two independent experiments.

### 3.3. Jump Dilution Assay

Jump dilution assays were performed according to a previously published protocol [33]. Initially, HDAC1 (14 ng/µL) was pre-incubated with test compounds (5× or 10× IC_50_) or vehicle in an “incubation mix” in assay buffer in PCR tubes for 60 min at room temperature. The “incubation mixtures” were then diluted 1:100 with either assay buffer or inhibitor solution at the original inhibitor concentration. Subsequently, 20 µL ZMAL substrate solution (250 µM) in assay buffer and 10 µL trypsin solution (1000 ng/µL) in trypsin buffer were added to a final volume of 100 µL. Fluorescence (excitation: 355 nm, emission: 460 nm) was measured continuously for 60 min at 28 °C at a Spark microplate reader (Tecan Group AG, Maennedorf, Switzerland). Compounds were tested in at least two independent experiments. For HDAC2, test compounds were initially incubated with HDAC2 (9.5 ng/µL) at 28 °C. ZMAL was used in a final concentration of 15 µM and trypsin in a concentration of 80 ng/µL.

### 3.4. CRISPR/Cas9-Mediated Gene Editing of HEK293-LgBiT Cells

A HiBiT-tag was engineered at the C-terminus of HDAC2 in HEK293 LgBiT stable cells (Promega, Madison, WI, USA; #N2672). HDAC2-HiBiT cells were generated by electroporation of ribonucleoprotein (RNP) complexes using a GenePulser Xcell electroporation device (BioRad, Feldkirchen, Germany). The targeting strategy including crRNA and ssODN sequences was adopted from Schwinn et al. [50]. For ribonucleoprotein (RNP) formation, HDAC2-crRNA and tracrRNA (IDT, Leuven, Belgium) were combined in IDTE buffer (IDT) to a final concentration of 100 μM and annealed (95 °C, 5 min; cool down to room temperature with −0.2 °C/s). For RNP assembly, 5 μM high-fidelity Cas9 protein (IDT) and 6 μM cr/tracrRNA were combined in PBS to a final volume of 17 μL and incubated for 20 min at room temperature. Subsequently, 8 μL of 100 μM ssODN were added. For electroporation, 2 × 10^6^ LgBiT-HEK293 cells were pelleted by centrifugation (800 rpm, 7 min), resuspended in 80 μL PBS and incubated for 5 min on ice. Cells were supplemented with 20 μL transfection mix, transferred to a pre-cooled 2 mm cuvette and electroporated (250 V, 2.0 ms pulse width, 1 pulse). Subsequently, cells were seeded on a T150 flask and incubated for 48 h in a tissue culture incubator (37 °C, 6% CO_2_). To generate monoclonal cell lines, single cells of the bulk population were seeded in 96-well plates by limiting dilution cloning. Established monoclonal cell lines were genotyped by PCR. PCR reactions were assembled according to the manufacturers protocol of the Platinum Taq DNA Polymerase (Thermo Fisher, Waltham, MA, USA; #10966) using gene-specific primers. Correct integration of the HiBiT tag was verified by Sanger sequencing (Eurofins, Ebersberg, Germany) of purified PCR products (NucleoSpin Gel and PCR clean up Kit, Macherey-Nagel, Düren, Germany).

### 3.5. Cloning of Expression Plasmids

A nucleotide sequence encoding human HDAC2 (UniProt ID Q92769) was purchased in the form of a DNA string (ThermoFisher Scientific Inc., Waltham, MA, USA) and inserted into the pDONR221 vector by a BP reaction mix using Gateway cloning strategy (Invitrogen, Thermo Fisher Scientific Inc., Waltham, MA, USA, #12535-019). The HDAC2 gene was transferred by LR reaction into a pDEST_MM345 destination vector resulting in the pEXP345_HDAC2 expression construct, where NanoLuc luciferase (NLuc) is fused to the HDAC2 C terminus. The pEXP345_HDAC2 expression plasmid was isolated using QIAGEN Plasmid Midi Kit (Qiagen, Hilden, Germany, #12143) and the sequence verified by Sanger sequencing.

### 3.6. Establishing HDAC2-NLuc-Overexpressing Cells

HEK293T cells were seeded onto a 6-well plate 24 h prior transfection. Transfection mixture was prepared by mixing 2 µg pEXP345_HDAC2 expression vector with 200 µL buffer and 8 µL JetPRIME tranfection reagent (Polyplus, Illkirch, France, #101000015) and incubated 8 min at RT. Transfection complexes were added to the cells and 4 h later cultivation media was exchanged with fresh medium. Selection of transfected cells started 48 h after transfection using hygromycin B (InvivoGen, San Diego, CA, USA, #ant-hg-1) at final concentration 400 µg/mL. In three weeks, single cell clones overexpressing HDAC2-NLuc were derived and established in cell culture media containing selection antibiotics.

### 3.7. NanoBRET Saturation Binding Assay

Saturation binding experiments were performed according to a previously published protocol with minor modifications [49]. Either 34 µL (non-permeabilized mode) or 30 µL (permeabilized mode) of a cell suspension in assay buffer (Opti-MEM^®^ Gibco, Thermo Fisher Scientific, Waltham, MA, USA; #11058-021) were pipetted to a white nonbinding 384-well microplate (PerkinElmer, Waltham, MA, USA; #6057480), resulting in 6800 cells/well. The fluorescent ligand **FFE2022** [49] was first serially diluted in DMSO and subsequently diluted 1:100 in assay buffer. Then, 2 µL of these dilutions and either 4 µL assay buffer or vorinostat (200 µM in assay buffer) were added to the cells to determine total binding and nonspecific binding, respectively. For experiments with permeabilized cells, 4 µL digitonin was added at a final concentration of 50 ng/µL. Permeabilized cells were incubated for 30 min at room temperature, whereas non-permeabilized cells were incubated at 37 °C and 5% CO_2_ for 2 h. After incubation, 20 µl NanoBRET^TM^ Nano-Glo^®^ substrate solution was added (Promega, Madison, WI, USA; #N1571; final dilution 1:2000). BRET signals were measured at 465/20 nm (donor) and 615/20 nm (acceptor) with a Tecan Spark plate reader (Tecan Group AG, Maennedorf, Switzerland). BRET ratios were calculated in milliBRET Units (mBU). Specific binding was determined by subtracting the non-specific binding from the total binding, and tracer affinity was determined using the one-site equation with GraphPad Prism 8.0 (San Diego, CA, USA).

### 3.8. NanoBRET Displacement Assay

Displacement assays were performed according to a previously published protocol with minor modifications [49]. First, 4 µL of fluorescent ligand **FFE2022** (final concentration: 0.5 µM) were added to 32 µL of cell suspension (6800 cells/well) in white non-binding 384-well microplates (PerkinElmer, Waltham, MA, USA). The test inhibitor was serially diluted in DMSO, followed by an additional 1:40 dilution in assay buffer. Then, 4 µL of these dilutions were added to the wells. Plates were incubated for 2 h at 37 °C and 5% CO_2_. Then, 20 µL substrate solution was added (Promega, Madison, WI, USA; #N1571; final dilution 1:2000). BRET signals were measured at 465/20 nm (donor) and 615/20 nm (acceptor) with a Tecan Spark plate reader (Tecan Group AG, Maennedorf, Switzerland). BRET ratios were calculated in milliBRET Units (mBU). EC_50_ values were determined by generating normalized dose–response curves using the four-parameter logistic equation (GraphPad Prism 8.0, San Diego, CA, USA). Data was normalized to untreated cells and a full inhibition control (inhibition at 20 µM vorinostat). All compounds were tested in duplicates, and EC_50_ values were calculated from at least three independent experiments. For experiments with HEK293 HDAC2-NLuc cells only 5000 cells/well were seeded, and the substrate was used in a final dilution of 1:5000.

### 3.9. NanoBRET Residence Time Assay

NanoBRET Residence Time Assay was performed according to a Promega protocol with minor modifications [65]. A cell suspension of 1.5 mL with a density of 0.24 × 10^6^ cells/mL in assay buffer was treated with the test inhibitors at a concentration corresponding to a multiple of the respective EC_50_ values. The maximum applied concentration was 100 µM. The samples were incubated at 37 °C and 5% CO_2_ for at least 2 h with a loosened cap. The cells were then washed once with 1.5 mL assay buffer to remove unbound inhibitor. Subsequently, 22 µL cell suspension, 6 µL of the fluorescent ligand **FFE2022** (final concentration: 1 µM), 6 µL digitonin (final concentration: 50 ng/µL), and 20 µL substrate solution (Promega, Madison, WI, USA; #N1571; final dilution 1:2000) were pipetted to a white 384-well microplate (PerkinElmer, Waltham, MA, USA). BRET signals were measured at 465/20 nm (donor) and 615/20 nm (acceptor) with a Tecan Spark plate reader (Tecan Group AG, Maennedorf, Switzerland) for at least 90 min. BRET ratios were calculated in milliBRET Units (mBU). The data were normalized to the highest signal of untreated cells, considering 100% BRET, and the lowest signal of cells treated with trapoxin A, representing 0% BRET. The experiment was repeated at least three times. For experiments with HEK293 HDAC2-NLuc cells, the substrate was used in a final dilution of 1:5000.

### 3.10. Immunoblot Wash Out Experiments

Immunoblot analysis was performed according to a previously published protocol [33]. HEK293-LgBiT cells (1 × 10^6^ cells for 24 h; 0.2 × 10^6^ cells for 72 h) were seeded into T25 cell culture flasks and were immediately treated with 10 µM HDAC inhibitor or vehicle (DMSO). After incubation, the cell culture media was removed, and the bottom of the flask was gently washed with 1 mL PBS. Subsequently, cells were cultured in a fresh inhibitor-free medium for either 0 h, 1 h, 3 h, or 6 h before lysis. For cell lysis, the cells were washed with PBS, detached with a scraper, and centrifuged at 1200 rpm for 4 min at 4 °C. The cell pellet was resuspended in 100 µL of freshly prepared lysis buffer and incubated on ice for 30 min. The lysis buffer consisted of Invitrogen cell extraction buffer (Thermo Fisher Scientific Inc., Waltham, MA, USA; #FNN0011), supplemented with 0.1 mM PMSF (Sigma-Aldrich, St. Louis, MO, USA; #10837091001) and HaltTM Protease Inhibitor (100×) (Life Technologies GmbH Carlsbad, CA, USA; #78429). Finally, the samples were centrifuged at 14,000 rpm and 4 °C for 30 min and the supernatant was collected. Protein concentration was determined with a BCA protein assay kit (Thermo Fisher Scientific Inc., Waltham, MA, USA; #23225). For SDS-PAGE, lysates were mixed with Laemmli sample buffer 2× concentrate (Sigma-Aldrich, St. Louis, MO, USA; #S3401-10VL) to a protein concentration of 1.25 µg/µL and denatured at 95 °C for 5 min. Proteins were separated on a 2–20% Mini-PROTEAN TGX stain-free gels (Bio-Rad, Hercules, CA, USA; #456809) at 200 V for 45 min. Proteins were then transferred to a Trans-Blot Turbo^®^-PVDF membrane (Bio-Rad, Hercules, CA, USA; #1704156) with the Trans-Blot Turbo Transfer System (Bio-Rad, Hercules, CA, USA; #1704150) at 25 V for 5 min. Alternatively, proteins were transferred by conventional wet transfer according to a previously published protocol [66]. The membranes were blocked with a solution of 5% milk powder in TBST (Tris-buffered saline-Tween 20 0.2%) for 60 min at room temperature and washed three times for 10 min each with TBST. Primary antibodies were applied overnight at 4 °C. For detection of acetylated H3, an anti-acetyl-histone H3 (lys9/Lys14) was used (Cell Signaling Technology, Denver, MA, USA; 1:2000 dilution; #9677). GAPDH was detected using a rabbit anti-GAPDH antibody (Cell Signaling Technology, Denver, MA, USA: 1:8000 dilution; #2118). After several washing steps, the membranes were incubated for 1.5 h at room temperature with HRP-conjugated secondary antibodies: anti-mouse IgGκ binding protein (Santa Cruz, Dallas, TX, USA; 1:10,000 dilution; #sc-516102) and anti-rabbit IgG antibody (R&D Systems, Inc., Minneapolis, MN, USA; 1:10,000 dilution; #HAF008). Protein bands were detected via chemiluminescence using Clarity Western ECL substrate (Bio-Rad, Hercules, CA, USA; #1705061). Detection and Analysis were performed with ChemiDox XRS+ System (Bio-Rad, Hercules, CA, USA; #1708265) and Image Lab software v. 6.1 (Bio-Rad, Hercules, CA, USA).

## 4. Conclusions

This work provides a comprehensive kinetic and cellular characterization of selected class I HDAC inhibitors using complementary biochemical and NanoBRET-based live-cell approaches. By comparing data from 100-fold jump dilution, NanoBRET residence time, and cellular washout experiments, we delineated distinct dissociation behaviors among inhibitors with similar potencies in end-point assays. Vorinostat displayed rapid, reversible binding consistent with fast-*on*/fast-*off* kinetics, whereas tucidinostat exhibited intermediate slow dissociation behaviors. In contrast, TNG260 and trapoxin A demonstrated markedly prolonged target engagement in cells, indicating tight-binding characteristics. Comparison of conventional and split NanoLuc-based NanoBRET assays revealed that endogenous HiBiT tagging of HDAC2 more accurately reflects native target engagement than overexpression-based systems. These findings underscore the importance of evaluating inhibitor kinetics under physiologically relevant conditions.

Collectively, our results indicate that dissociation kinetics represent an important contributing factor to pharmacological efficacy and may complement traditional potency-based assessments. The prolonged residence times of TNG260 and trapoxin A suggest potential advantages for achieving sustained target modulation with reduced systemic exposure. Furthermore, the observed differences in the kinetics of class I-selective inhibitors are valuable for the rational design of next-generation HDAC inhibitors with improved selectivity for specific isoforms and complexes, optimized residence times, and enhanced therapeutic safety profiles.

## Figures and Tables

**Table 1:** Inhibitory activity against HDAC1, HDAC2, and HDAC3/NCoR in the biochemical HDAC inhibition assay and against HDAC2 in split NLuc-based NanoBRET assays with LgBiT-expressing HEK293 HDAC2-HiBiT cells.

Inhibitor	HDAC Inhibition Assay IC_50_ [µM]			NanoBRET EC_50_ [µM]
	HDAC1 ^[a,b]^	HDAC2 ^[a,b]^	HDAC3/NCoR ^[a,b]^	HDAC2 ^[c]^
vorinostat	0.11 ± 0.01	0.21 ± 0.02	0.10 ± 0.02	0.38 ± 0.07
panobinostat	0.0031 ± 0.0013	0.0038 ± 0.0007	0.0011 ± 0.0004	0.0034 ± 0.0004
tucidinostat	0.24 ± 0.02	0.24 ± 0.04	0.10 ± 0.02	1.4 ± 0.3
entinostat	0.43 ± 0.06 [37]	0.35 ± 0.04 [37]	0.31 ± 0.01 [37]	1.8 ± 1.4
VK-1	0.069 ± 0.010 [37]	0.13 ± 0.02 [37]	0.31 ± 0.01 [37]	0.74 ± 0.19
trapoxin A	0.00029 ± 0.00005	0.00027 ± 0.00006	0.00051 ± 0.00018	0.00037 ± 0.00010
TNG260	0.033 ± 0.002	0.086 ± 0.016	4.5 ± 0.1	0.012 ± 0.005
tubastatin A	5.1 ± 1.3	8.4 ± 1.8	6.4 ± 0.4	1.7 ± 1.1

^[a]^ IC_50_ values are reported as mean ± standard deviation (SD) from at least two independent experiments. ^[b]^ Pre-incubation of enzyme and inhibitor: 1 h at RT. ^[c]^ EC_50_ values are reported as mean ± standard error of the mean (SEM) from at least three independent experiments.

**Table 2:** Inhibitory activity in NanoBRET assays with HDAC1-NLuc, HDAC2-NLuc, and HDAC2-HiBiT fused cells.

Inhibitor		EC_50_ [µM]	
	HDAC1-NLuc ^[a]^	HDAC2-NLuc ^[a]^	HDAC2-HiBiT ^[a]^
vorinostat	0.81 ± 0.41	0.98 ± 0.18	0.38 ± 0.07
tucidinostat	18 ± 7	0.69 ± 0.09	1.4 ± 0.3
trapoxin A	0.00024 ± 0.00010	0.00032 ± 0.00032	0.00037 ± 0.00010
TNG260	0.30 ± 0.03	0.045 ± 0.008	0.012 ± 0.005
tubastatin A	72 ± 27	21 ± 2	1.7 ± 1.1

^[a]^ EC_50_ values are reported as mean ± standard error of the mean (SEM) from at least three independent experiments.

## Data Availability

The original contributions presented in this study are included in the article/Supplementary Materials. Further inquiries can be directed to the corresponding author.

## Supplementary Materials

The following supporting information can be downloaded at https://www.mdpi.com/article/10.3390/ijms27073036/s1.

## Abbreviations

The following abbreviations are used in this manuscript:

CoREST	repressor element-1 silencing transcription co-repressor
DMD	*Duchenne* muscular dystrophy
DMSO	dimethylsulfoxide
EC_50_	half maximal effective concentration
FDA	Food and Drug Administration
HAT	histone acetyltransferase
HDAC	histone deacetylase
HDACi	histone deacetylase inhibitor
HiBiT	high-affinity binary technology
IC_50_	half maximal inhibitory concentration
*K*_D_	dissociation constant
LgBiT	large binary technology subunit
MiDAC	mitotic deacetylase complex
MIER	mesoderm induction early response
NanoBiT	NanoLuc Binary Technology
NanoBRET	NanoLuciferase bioluminescent resonance energy transfer
NCoR1	nuclear receptor co-repressor 1
NCoR2	nuclear receptor co-repressor 2
NLuc	NanoLuciferase
NMPA	National Medical Products Administration
NuRD	nucleosome remodeling deacetylase
PTM	post-translational modifications
RERE	arginine-glutamic acid dipeptide repeats
Sin3A	SWI-independent-3A
SMRT	silencing mediator of retinoic acid and thyroid hormone receptor
tpx A	trapoxin A
tuc.	tucidinostat
vor.	vorinostat
ZBG	zinc-binding group
