# Supplementary material for: Cellular Target Engagement and Dissociation Kinetics of Class I-Selective Histone Deacetylase (HDAC) Inhibitors
Source: Int J Mol Sci. 2026 Mar 26;27(7):3036. doi: 10.3390/ijms27073036 (PMC13073407; doi:10.3390/ijms27073036)
Supplement: Supplementary file 1 [file ijms-27-03036-s001.zip › ijms-4128870-supplementary.pdf]

Supplementary Information

Cellular Target Engagement and Dissociation Kinetics of Class I Selective HDAC Inhibitors

Irina Honin[a], Zora Novakova[b], Felix Feller[a], Simon Schneider[c], Linda Schäker-Hübner[a], Cyril Bařinka[b], Finn K. Hansen [a]*

[a]Department of Pharmaceutical and Cell Biological Chemistry, Pharmaceutical Institute, University of Bonn, An der Immenburg 4, 53121 Bonn, Germany.

[b]Institute of Biotechnology of the Czech Academy of Sciences, BIOCEV, Prumyslova 595, 252 50 Vestec, Czech Republic.

[c]Bonn Technology Campus, Core Facility 'Gene-Editing', Medical Faculty, University of Bonn, 53127 Bonn, Germany.

* Correspondence to: finn.hansen@uni-bonn.de

Prof. Dr. Finn K. Hansen, Pharmaceutical and Cell Biological Chemistry, Pharmaceutical Institute, University of Bonn, An der Immenburg 4, 53121 Bonn, Germany. Tel.: (+49) 228 73 5213. Fax: (+49) 228 73 7929. E-mail: finn.hansen@uni-bonn.de.

TABLE OF CONTENTS

1. SUPPLEMENTARY FIGURES S3

1. SUPPLEMENTARY FIGURES

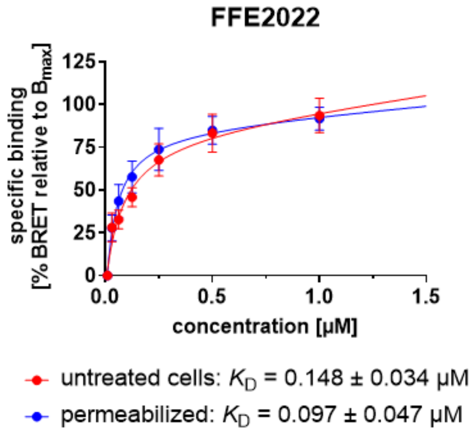

Figure S1. Binding affinity of fluorescent ligand **FFE2022**: NanoBRET curves displaying the affinity of the fluorescent ligand **FFE2022** in untreated (red) and permeabilized (digitonin-treated, 50 ng/µL; blue) LgBiT-expressing HEK293 HDAC2-HiBiT cells. K_D values are reported as mean ± standard error of the mean (SEM) from at least two independent experiments.

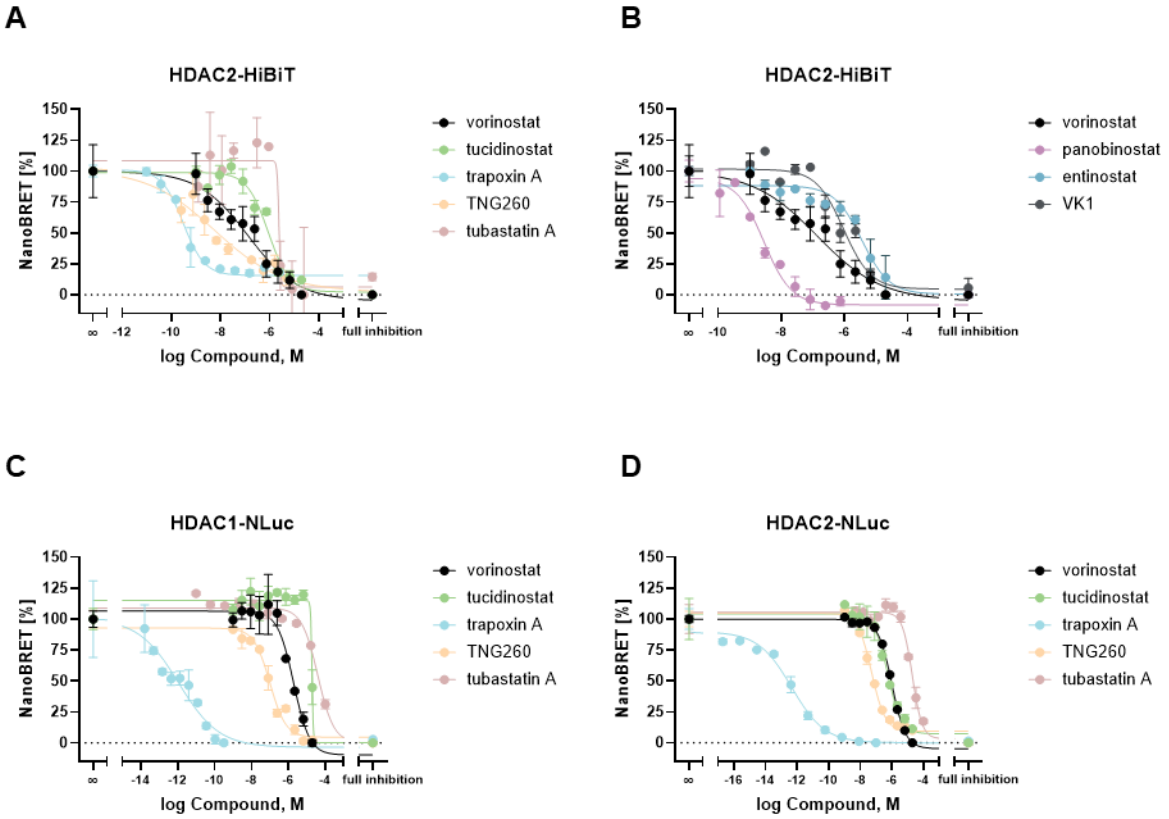

Figure S2. Cellular HDAC1 and HDAC2 NanoBRET engagement assays. For the NanoBRET target engagement assay, HEK293 cells stably expressing HDAC2-HiBiT (**A**/**B**), HDAC1-NLuc (**C**), and HDAC2-NLuc cells were used (**D**). Representative dose-response curves from at least three independent experiments are shown (mean ± standard error of the mean (SEM)).

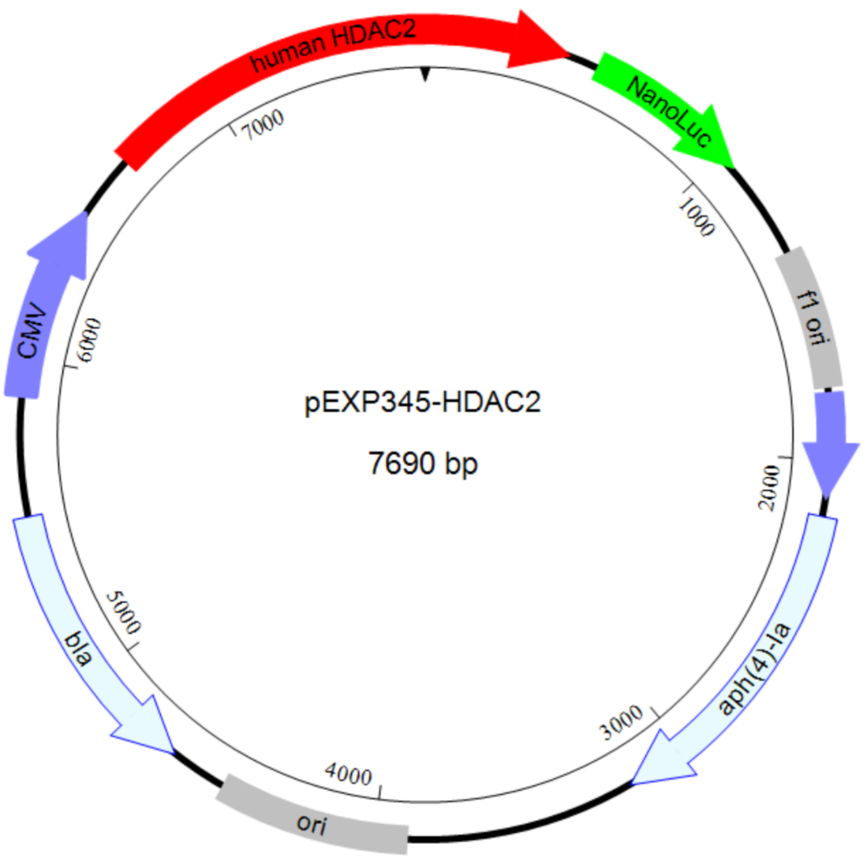

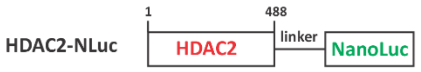

MAYSQGGGKKKVCYYYDGDIGNYYYGQGHPMKPHRIRMTHNLLLNYGLYRKMEIYRPHKATAEE
MTKYHSDEYIKFLRSIRPDNMSEYSKQMQRFNVGEDCPVFDGLFEFCQLSTGGSVAGAVKLNRQ
QTDMAVNWAGGLHHAKKSEASGFCYVNDIVLAILELLKYHQRVLYIDIDIHHGDGVEEAFYTTDRVM
TVSFHKYGEYFPGTGDLRDIGAGKGKYYAVNFPMRDGIDDESYGQIFKPIISKVMEMYQPSAVVLQ
CGADSLSGDRLGCFNLTVKGHAKCVEVVKTFNLPLLMLGGGGYTIRNVARCWTYETAVALDCEIPN
ELPYNDYFEYFGPDFKLHISPSNMTNQNTPEYMEKIKQRLFENLRMLPHAPGVQMQAIPEDAVHE
DSGDEDGEDPDKRISIRASDKRIACDEEFSDSEDEGEGGRRNVADHKKGAKKARIEEDKKETEDK
KTDVKEEDKSKDNSGEKTDTKGTKSEQLSNPPSFLVQSGYIQHSGGRSSGSGSTSGSGKTGVFT
LEDFVGDWRQTAGYNLDQVLEQGGVSSLFQNLGVSVTPIQRIVLSGENGLKIDIHVIIPYEGLSGDQ
MGQIEKIFKVVYPVDDHHFKVILHYGTLVIDGVTPNMIDYFGRPYEGIAVFDGKKITVTGTLWNGNKII
DERLINPDGSLLFRVTINGVTGWRLCERILA

Figure S3. Plasmid map and sequence for HDAC2. This plasmid was used for the expression of HDAC2 in mammalian HEK293T cells. The HDAC sequence is C-terminally fused to NLuc.

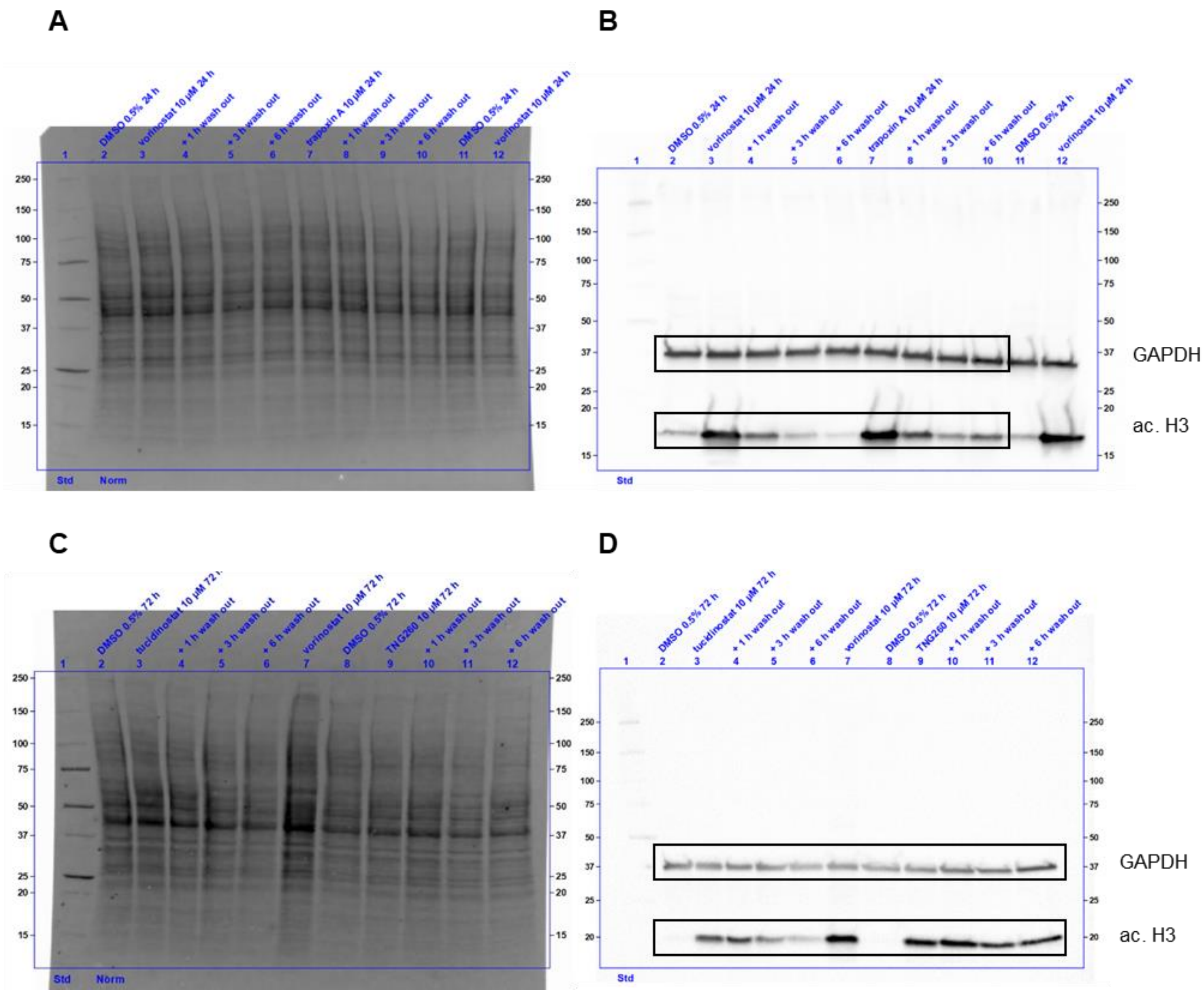

Figure S4. Representative immunoblot images of stain-free total protein blots after transfer (**A**/**C**) and the corresponding immunodetection of GAPDH and ac. H3 (K9/K14) for vorinostat and trapoxin A (**B**), and for tucidinostat and TNG260 (**D**).
